# Non-invasive hepatic fat quantification: Can multi-echo Dixon help?

**DOI:** 10.1590/0100-3984.2023.0125

**Published:** 2024-05-07

**Authors:** Akarshi Gupta, Rashmi Dixit, Anjali Prakash

**Affiliations:** 1 Department of Radiodiagnosis, Lok Nayak Hospital - Maulana Azad Medical College, New Delhi, India

**Keywords:** Fatty liver/diagnosis, Magnetic resonance imaging/methods, Magnetic resonance spectroscopy, Non-alcoholic fatty liver disease/diagnosis, Fígado gorduroso/diagnóstico, Ressonância magnética/métodos, Espectroscopia de ressonância magnética, Hepatopatia gordurosa não alcoólica/diagnóstico

## Abstract

**Objective:**

To evaluate the diagnostic accuracy of multi-echo Dixon magnetic resonance imaging (MRI) in hepatic fat quantification, in comparison with that of magnetic resonance spectroscopy (MRS), on 3.0-T MRI.

**Materials and Methods:**

Fifty-five adults with no known liver disease underwent MRI in a 3.0-T scanner for determination of the hepatic fat fraction, with two techniques: multi-echo Dixon, in a manually drawn region of interest (ROI) and in the entire liver parenchyma (automated segmentation); and MRS. The diagnostic accuracy and cutoff value for multi-echo Dixon were determined, with MRS being used as the reference standard.

**Results:**

The mean fat fraction obtained by multi-echo Dixon in the manually drawn ROI and in the entire liver was 5.2 ± 5.8% and 6.6 ± 5.2%, respectively, whereas the mean hepatic fat fraction obtained by MRS was 5.7 ± 6.4%. A very strong positive correlation and good agreement were observed between MRS and multi-echo Dixon, for the ROI (r = 0.988, r^2^ = 0.978, *p* < 0.001) and for the entire liver parenchyma (r = 0.960, r^2^ = 0.922, *p* < 0.001). A moderate positive correlation was observed between the hepatic fat fraction and body mass index of the participants, regardless of the fat estimation technique employed.

**Conclusion:**

For hepatic fat quantification, multi-echo Dixon MRI demonstrated a very strong positive correlation and good agreement with MRS (often considered the gold-standard noninvasive technique). Because multi-echo Dixon MRI is more readily available than is MRS, it can be used as a rapid tool for hepatic fat quantification, especially when the hepatic fat distribution is not homogeneous.

## Abstract (pt)

**Objetivo:**

Avaliar a acurácia diagnóstica da técnica multieco Dixon na quantificação da gordura hepática em comparação com a espectroscopia por ressonância magnética (ERM), em exames de RM 3.0-T.

**Materiais e Métodos:**

Cinquenta e cinco participantes adultos sem doença hepática conhecida foram submetidos a RM 3.0-T para determinação da fração de gordura hepática, usando duas técnicas: multieco Dixon (em ROI desenhada manualmente e em segmentação automatizada para todo o parênquima hepático) e ERM. A precisão diagnóstica e o valor de corte para multieco Dixon foram determinados usando a ERM como padrão de referência.

**Resultados:**

A fração de gordura média usando multieco Dixon na ROI desenhada manualmente e na segmentação automatizada do fígado inteiro foi 5,2 ± 5,8% e 6,6 ± 5,2%, respectivamente. A fração de gordura hepática média usando ERM foi 5,7 ± 6,4%. Correlação positiva muito alta e forte concordância foram observadas entre ERM e multieco Dixon, tanto para ROI (r = 0,988, r^2^ = 0,978, *p* < 0,001) quanto para todo o parênquima hepático (r = 0,960, r^2^ = 0,922, *p* < 0,001). Correlação positiva moderada foi observada entre a fração de gordura hepática e o índice de massa corpórea dos participantes usando ambas as técnicas de estimativa de gordura.

**Conclusão:**

Multieco Dixon demonstrou correlação positiva muito alta e concordância com a ERM (muitas vezes considerada padrão de referência não invasivo) para quantificação de gordura hepática. Uma vez que o multieco Dixon está mais prontamente disponível do que a ERM, pode ser usado como uma ferramenta rápida para a quantificação da gordura hepática, especialmente na distribuição não homogênea da gordura.

## INTRODUCTION

The liver is responsible for a variety of key functions in human physiology^(1)^, such as lipid and carbohydrate homeostasis; detoxification of blood; removal of infectious agents via Kupffer cells; and maintenance of iron homeostasis. Any alteration in the metabolism of fatty acids can lead to their accumulation within hepatocytes, causing oxidative stress, which in turn leads to activation of stellate cells and hepatocellular injury, thereby impairing hepatic function. In a normal liver, fatty changes are present in ≤ 5% of hepatocytes. Excessive deposition of triglycerides within hepatocytes results in fatty liver, also known as hepatic steatosis^(2)^.

Recently, steatotic liver disease (SLD) has been classified into subtypes, as follows^(3)^: metabolic dysfunction-associated steatotic liver disease (MASLD); alcohol-associated liver disease; MASLD and increased alcohol intake; other specific etiology SLD; and cryptogenic SLD. In many parts of the world, the progressive adoption of a sedentary lifestyle with excess caloric intake has led to an increase in the prevalence of obesity and MASLD, even among so-called healthy individuals with no comorbidities^(4)^. In 20-30% of patients with MASLD, there is progression to metabolic dysfunction-associated steatohepatitis, which is characterized by inflammation and ballooning of hepatocytes, leading to cirrhosis in 5% of such patients. Even in the absence of cirrhosis, fatty liver disease is a risk factor for the development of hepatocellular carcinoma, as well as renal and cardiovascular comorbidities^(5)^.

Insulin resistance and metabolic syndrome are strongly associated with MASLD and contribute to the development of steatohepatitis^(6)^. Because this spectrum of disorders is often asymptomatic or silent and may affect young individuals, lean individuals, and even children or adolescents, vigilance and early detection of hepatic steatosis can help improve clinical outcomes^(6-8)^. However, no surveillance guidelines exist and the use of imaging is largely based on clinician recommendations.

The gold standard for the characterization of hepatic steatosis is percutaneous image-guided biopsy, which is an invasive procedure and provides the fat fraction for only an extremely small part of the liver and therefore does not account for the heterogeneous nature of fat deposition. Non-invasive detection and quantification of hepatic steatosis can be achieved by using various imaging modalities, including ultrasound, computed tomography (CT) and magnetic resonance imaging (MRI). Conventional ultrasound, albeit widely used, has poor sensitivity for identifying low-grade steatosis^(9)^. Multiple quantitative ultrasound techniques, based on acoustic parameters such as the attenuation coefficient, backscatter coefficient, speckle patterns, and speed of sound, have been developed^(10)^. Among those, the most widely studied is the controlled attenuation parameter, which is acquired from raw radiofrequency data during ultrasound-based vibration-controlled transient elastography^(10)^. A CT scan also has poor sensitivity and specificity for mild steatosis, as well as exposing subjects to ionizing radiation^(11)^. In recent years, MRI and magnetic resonance spectroscopy (MRS) have emerged as accurate methods to quantify liver triglyceride concentration based on the difference in resonant frequency between fat and water^(12)^. The fat content estimated by using MRI-based techniques has been found to show an excellent correlation with the histological grade of hepatic steatosis. The present study entails detection and quantification of hepatic steatosis in a 3.0-T MRI scanner using two MRI techniques: a chemical shift-based technique known as multi-echo Dixon; and MRS. The latter is widely considered the reference standard for non-invasive hepatic fat detection^(13,14)^. The objectives of this study were to assess the prevalence of hepatic steatosis among participants with no known liver disease and to evaluate the diagnostic accuracy of multi-echo Dixon in hepatic fat quantification in comparison with that of MRS.

## MATERIALS AND METHODS

### Participants

This cross-sectional study was approved by the local institutional review board and research ethics committee. All participants gave written informed consent. A total of 58 consecutive individuals ≥ 18 years of age and without known liver disease were initially recruited for this study. The exclusion criteria were having a personal or family history of diabetes mellitus; consuming an excessive quantity of alcohol (defined as > 60 g/day for men and > 20 g/day for women); having had a blood transfusion; being pregnant; having undergone a surgical procedure involving the liver; having any abnormal random blood sugar levels; having had an abnormal liver function test result; having had abnormal findings on prior hepatic imaging; and having used oral contraceptives, lipid-lowering drugs, antituberculosis drugs, corticosteroids, antihypertensive drugs, or antidiabetic medication. Individuals in whom MRI was contraindicated were also excluded. Only three volunteers were excluded: two because they were unable to perform a breath-hold for a sufficient length of time; and one because of claustrophobia. Therefore, the final sample comprised 55 subjects. The age, gender, weight, and height of the participants were recorded. Body mass index (BMI) was calculated as weight in kilograms divided by height in meters squared (kg/m^2^).

### Imaging technique

In all subjects, MRI of the abdomen was performed in a 3.0-T scanner (Magnetom Skyra; Siemens Healthineers, Erlangen, Germany) using a body coil that covered the region from just below the level of the nipple to the umbilicus. To localize the area of interest, axial and coronal T2-weighted half-Fourier acquisition single-shot turbo spin-echo images were acquired. This was followed by application of a specialized package (LiverLab, with syngo MR E11 software; Siemens Healthineers) that includes three sequences: T1-weighted volumetric interpolated breath-hold examination (VIBE) screening Dixon (occasionally referred to as e-Dixon), VIBE multi-echo Dixon (occasionally referred to as q-Dixon), and breath-hold single-voxel high-speed T2-corrected multi-echo ^1^H MRS (HISTO).

**Figure 1:**
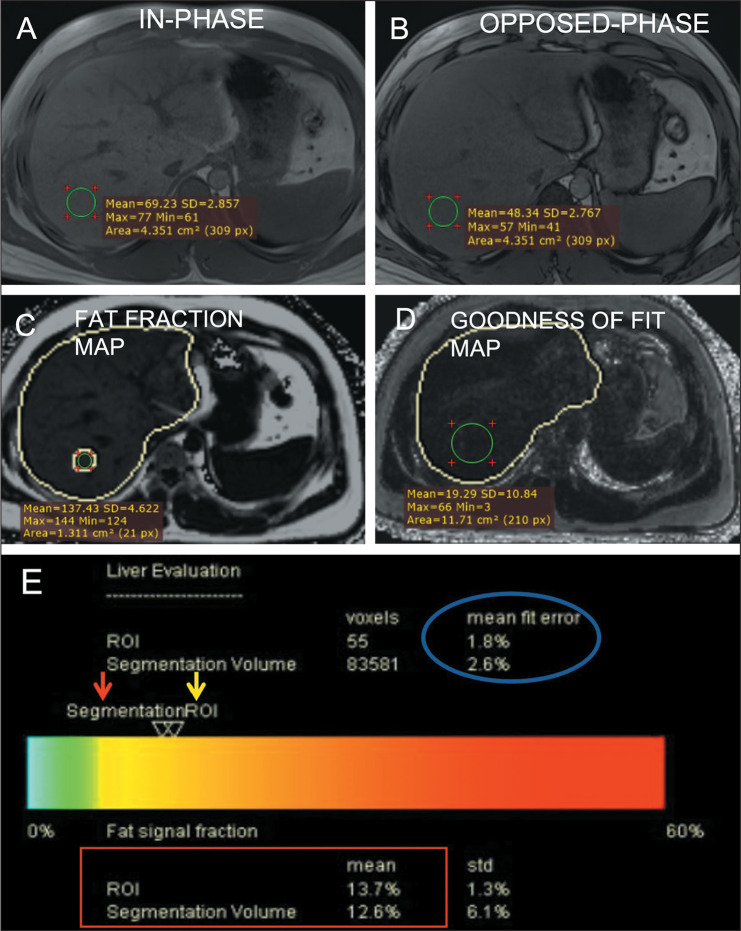
Hepatic fat estimation using Dixon sequences. In-phase (A) and opposed-phase (B) images provided by screening Dixon show a drop in signal intensity in the opposed phase images, indicating fat deposition. The mean signal intensity of an ROI drawn in a fat fraction map (C) multiplied by 0.1 gives the fraction of fat in that ROI. Similarly, the mean signal intensity value in a goodness-of-fit map (D) multiplied by 0.1 gives the fit error (which should be < 5% for reliable fat quantification). The report (E) provides the fat fraction in the form of a color bar and the numerical value (red box) in the ROI (yellow arrow) and entire liver as a whole (automated segmentation, red arrow).

**Figure 2:**
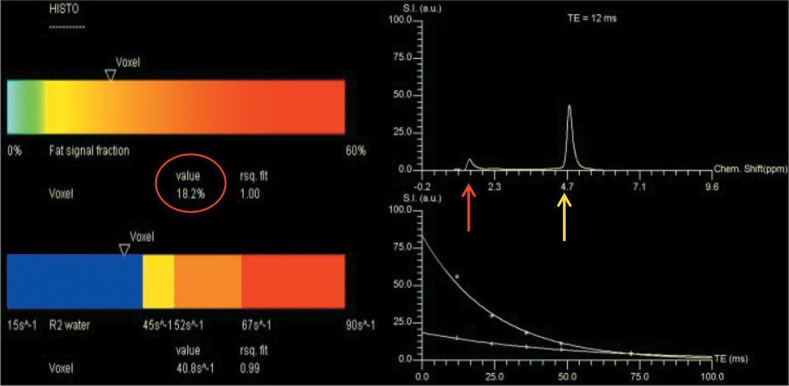
Hepatic fat estimation by MRS, showing the fat fraction in the voxel (18.2%, suggestive of steatosis, in this case), a good T2 relaxation curve for water and fat, with the spectral peak showing fat at 1.3 ppm (red arrow) and water at 4.7 ppm (yellow arrow).

After the subjects had been given the appropriate instructions regarding the breathing maneuvers, a T1-weighted VIBE screening Dixon sequence was acquired. That dual-echo three-dimensional sequence provided whole liver coverage and generated in-phase and opposed-phase images with the following imaging parameters: repetition time/first echo time/second echo time (TR/TE1/TE2), 3.97/1.29/2.52 ms; matrix, 195 × 320; slice thickness, 3.0 mm; and flip angle, 9°. In-phase and opposed-phase images obtained from the screening Dixon sequence were analyzed by visual assessment for fat deposition, as evidenced by a drop in signal intensity on the opposed-phase images. A VIBE multi-echo Dixon sequence was then acquired, with the following parameters: TR/TE1/TE2/TE3/TE4/TE5/TE6, 9.0/1.09/2.46/3.69/4.92/6.15/7.38 ms; matrix, 101 × 160; slice thickness, 3.5 mm; and flip angle, 4°. Eight series of images were generated by multi-echo Dixon sequence: water only; fat only; fat fraction; goodness-of-fit; R2* map; T2* map; water fraction; and evaluation report. The entire liver was outlined by automatic inline segmentation. The fat fraction was automatically corrected for T2* effects. Images were also evaluated to exclude fat-water swaps. This technique provides fat quantification through chemical shift imaging. The hepatic MRI proton density fat fraction (MRI-PDFF) value provided by multi-echo Dixon for automated segmentation of the entire liver parenchyma was recorded. A region of interest (ROI) was placed over the right lobe of the liver, excluding blood vessels, bile ducts, and the gall bladder, as well as being positioned to avoid cardiac pulsations. The fat fraction in this ROI was also recorded. To ensure that the acquisition provided a reliable fat estimation, the goodness-of-fit value adopted was < 5% for all acquisitions (Figure 1). Finally, a HISTO sequence was acquired. A single 3 × 3 × 3 cm^3^ voxel was co-localized at the same location as the ROI in the multi-echo Dixon sequence, with the axial, coronal, and sagittal planes being used as localizers. The sequence was acquired with a TR/TE1/TE2/TE3/TE4/TE5 of 2,200/12/24/36/48/72 ms. For quality control, an r^2^ (goodness of fit) of > 0.95 with good T2 relaxation curves for fat and water was ensured in all cases. A HISTO sequence gives a hepatic fat quantification value using MRS. The PDFF provided by MRS (MRS-PDFF) for the co-localized voxel was recorded (Figure 2).

To identify hepatic steatosis, we used a hepatic PDFF cutoff of > 5%, as suggested by Zhao et al.^(13)^. Thus, a hepatic fat fraction ≤ 5% was classified as normal.

The correlation and agreement between the hepatic MRI-PDFF obtained by multi-echo Dixon and the MRS-PDFF were evaluated, and a receiver operating characteristic (ROC) curve analysis was performed to calculate the clinical utility of and optimal cutoff values for multi-echo Dixon, using MRS as the reference standard. The correlation between BMI and the PDFF was also investigated, as was the association between gender and the presence of hepatic steatosis.

### Statistical analysis

Data were entered into MS Excel, and statistical analyses were performed with the IBM SPSS Statistics software package, version 25.0 (IBM Corp., Armonk, NY, USA). Continuous quantitative variables were summarized as mean ± standard deviation or as median (interquartile range), depending on the distribution of data. The correlation between the MRI-PDFF obtained by multi-echo-Dixon and the MRS-PDFF was determined by linear regression analysis. Bland-Altman analysis was used in order to evaluate agreement between the MRI-PDFF obtained by multi-echo-Dixon and the MRS-PDFF. For all statistical tests, values of *p* < 0.05 were considered statistically significant.

## RESULTS

Of the 55 adults evaluated, 23 were male and 32 were female. The mean age was 33.89 ± 11.9 years (range, 18-65 years), and the mean BMI was 25.33 ± 4.6 kg/m^2^ (range, 16.0-34.3 kg/m^2^). On the basis of the BMI and the Asian modification of the World Health Organization classification, 16 (29.1%) of the participants were classified as underweight or normal and 39 (70.9%) were classified as overweight or obese. All participants underwent multi-echo Dixon and MRS. Visual inspection of screening Dixon images revealed a drop in signal intensity on opposed-phase images in only 14 (25.5%) of the participants. We recorded the fat fraction values obtained by multi-echo Dixon (for the entire liver parenchyma and for the manually drawn ROI) and by MRS (Table 1).

**Table 1:** Hepatic fat fraction values and proportion of participants showing hepatic steatosis (PDFF > 5%) on multi-echo Dixon and MRS.

Measure	Hepatic fat fraction		
	Multi-echo Dixon		MRS
	Entire liver	ROI	
Mean ± SD	6.6 ± 5.2%	5.2 ± 5.8%	5.7 ± 6.4%
Range	1.8-22.5%	0.8-24.6%	0.8-25.9%
Median (IQR)	4.3% (5.05%)	2.3% (5.1%)	2.4% (5.5%)
Proportion of participants with a PDFF > 5%	43.6%	30.9%	32.7%

SD, standard deviation; IQR, interquartile range.

**Figure 3:**
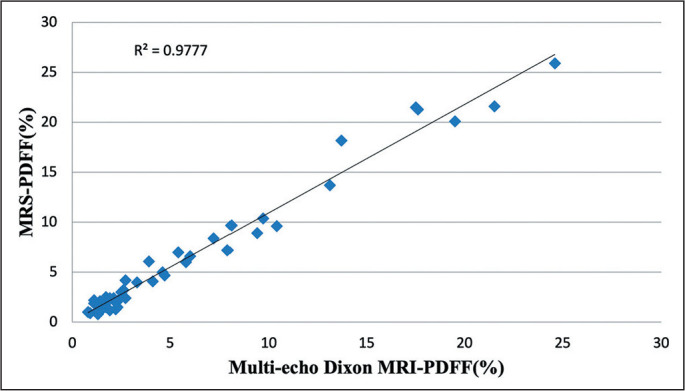
Scatter plot between the MRS-PDFF and the multi-echo Dixon MRI-PDFF for the ROI.

**Figure 4:**
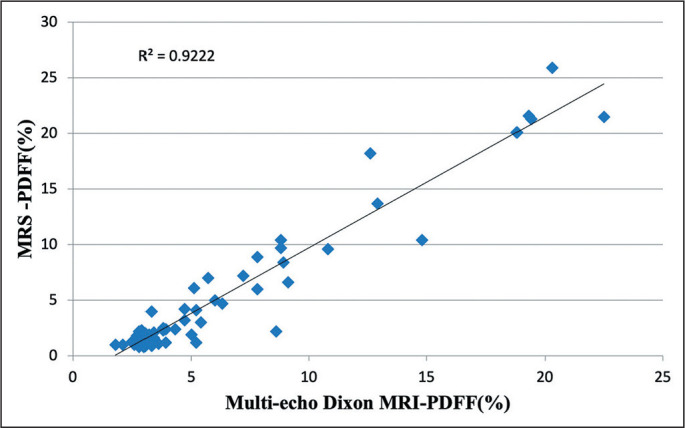
Scatter plot between the MRS-PDFF and the multi-echo Dixon-PDFF for the entire liver.

Hepatic steatosis (i.e., a fat fraction > 5%) was evident in 17 (30.9%) of the 55 participants when multi-echo Dixon was used for a manually drawn ROI, compared with 18 (32.7%) when MRS was used. The median and mean hepatic fat fraction values obtained by multi-echo Dixon were 4.3% and 6.6 ± 5.2%, respectively, for the entire liver parenchyma, compared with 2.3% and 5.2 ± 5.8%, respectively, for the manually drawn ROI. For the detection of hepatic steatosis (MRI-PDFF > 5%) in the manually drawn ROI, multi-echo Dixon was found to have a sensitivity, specificity, positive predictive value (PPV), negative predictive value (NPV), and accuracy of 94.4%, 100%, 100%, 97.4%, and 97.2%, respectively, compared with 100%, 83.8%, 75.0%, 100%, and 89.1%, respectively, for its detection in the liver parenchyma as a whole (by automated segmentation). As illustrated in Figures 3 and 4, the linear regression analysis revealed an excellent correlation between the MRI-PDFF obtained by using multi-echo Dixon and the MRS-PDFF, for the co-localized ROI (r = 0.988, r^2^ = 0.978, *p* < 0.001) and for the liver parenchyma as a whole (r = 0.960, r^2^ = 0.922, *p* < 0.001). The Bland-Altman analysis revealed strong agreement between the MRI-PDFF obtained by using multi-echo Dixon and the MRS-PDFF, with a bias (upper and lower limit of agreement) of 0.509 (2.62, -1.60) and -0.89 (3.03, -4.82), respectively.

Using the Youden Index, we calculated the optimal multi-echo Dixon-derived hepatic MRI-PDFF cutoff to detect hepatic steatosis to be 4.7% when measured in the ROI corresponding to the MRS voxel, with a sensitivity, specificity, PPV, NPV, and accuracy of 94.4%, 100%, 100%, 97.4%, and 98.2%, respectively. Similarly, the optimal threshold for detecting hepatic steatosis was calculated to be 5.4% when multi-echo Dixon-derived hepatic MRI-PDFF values were determined for the entire liver, with a sensitivity, specificity, PPV, NPV, and accuracy of 94.4%, 91.9%, 85.0%, 97.1%, and 92.7%, respectively.

Of the participants who were classified as underweight or normal weight (BMI < 22.9 kg/m^2^), none were found to have hepatic steatosis, which was seen in only one of the participants who was classified as overweight (BMI of 23-24.9 kg/m^2^). Among the 27 participants who were classified as obese (BMI > 25 kg/m^2^), 17 (63.0%) had hepatic steatosis when the MRS-PDFF was used for evaluation, compared with 16 (59.3%) when the multi-echo Dixon-derived MRI-PDFF was used. A moderate positive correlation was observed between BMI and the hepatic fat fraction, when multi-echo Dixon was used in the ROI (r = 0.661, r^2^ = 0.436; *p* < 0.001) and when MRS was used (r = 0.669, r^2^ = 0.447; *p* < 0.001).

In our study sample, hepatic steatosis was more common among the men than among the women (43.5% vs. 25.0%). However, that association did not reach the level of statistical significance (*p* > 0.05).

## DISCUSSION

In this study, we observed a very high positive correlation and strong agreement between the hepatic fat fraction obtained by multi-echo Dixon and that obtained by MRS. Our findings demonstrate the feasibility and potential clinical utility of multi-echo Dixon in quantifying hepatic steatosis.

We observed that, in all of our participants with hepatic steatosis, the drop in signal intensity on opposed phase images was uniform throughout the liver. That finding is in agreement with those of previous studies showing that, in individuals with hepatic steatosis, diffuse fat distribution is the most common pattern^(15)^.

We found that, on multi-echo Dixon, the fat fraction values obtained for the entire liver were slightly higher than were those obtained for the manually drawn ROI. Our observation was similar to that of Zhang et al.^(16)^, who reported higher fat fraction values for whole-liver segmentation than for an ROI in healthy individuals with mild hepatic steatosis. That could be due to the inclusion of periportal fat and fat in the intrahepatic fissure in the whole-liver segmentation.

The median and mean fat fraction values for the manually drawn ROI in the present study are similar to those reported by Kühn et al.^(17)^, for a population in Germany, among which the median MRI fat fraction for a manually drawn ROI was 3.9%. However, Hetterich et al.^(18)^, Patil et al.^(14)^, and Kuchay et al.^(19)^ reported mean fat fraction values of 9.2%, 8.65%, and 13.0% respectively, which can be attributed to the fact that the exclusion criteria applied were less rigid than those applied in our study.

The moderate positive correlation observed between BMI and liver fat fraction when either multi-echo Dixon ROI or MRS was used is similar to what was demonstrated by Hines et al.^(20)^ and Di Martino et al.^(21)^. It is generally accepted that a higher BMI, especially that indicative of obesity, is likely to be associated with fat deposition in the liver. The moderate positive correlation observed between BMI and hepatic fat in the present study could be due to the lack of a singular cause-and-effect relationship between obesity and hepatic fat, which limits the ability of anthropometric parameters to identify hepatic steatosis accurately.

In the present study, the proportion of participants found to have hepatic steatosis was higher among the men, although no statistically significant correlation was detected between hepatic steatosis and gender. Our findings are similar to those obtained by Yu et al.^(22)^ in a study of children with obesity in China. The authors found the prevalence of hepatic steatosis to be higher in the boys than in the girls (29.4% vs. 22.6%), although they also detected no significant correlation between hepatic steatosis and gender.

The optimal cutoff values for the detection of hepatic steatosis by multi-echo Dixon based on our ROC curve analysis are similar to those determined by Zhao et al.^(13)^, who found the optimal multi-echo Dixon cutoff values to be 5.1% for a manually drawn ROI and 5.4% for the entire liver parenchyma, using MRS as the reference standard.

In the present study, hepatic steatosis was diagnosed in 17 participants when multi-echo Dixon was used in a manually drawn ROI and in 18 participants when MRS was used. Therefore, if MRS is considered the reference standard in the absence of liver biopsy, multi-echo Dixon wrongly classified only one patient, in whom MRS showed the fat fraction to be 6.1%. The prevalence of hepatic steatosis among our study participants is in agreement with that reported by Szczepaniak et al.^(23)^, who retrospectively analyzed participants in the Dallas Heart Study with no known liver disease, using MRS for the estimation of hepatic steatosis, and found 33.6% to have elevated hepatic triglyceride content. In contrast, Rehm et al.^(6)^ found the prevalence of hepatic steatosis to be only 15% in a sample of adolescent girls and young women.

We detected a very strong correlation and good agreement between the multi-echo Dixon-derived MRI-PDFF for the manually drawn ROI and the MRS-PDFF. We also demonstrated a very strong correlation between the multi-echo Dixon-derived fat fraction for the entire liver parenchyma and that obtained by single-voxel MRS. Our findings are consistent with those of Zhao et al.^(13)^, Bashir et al.^(24)^, and Yokoo et al.^(25)^, all of whom used MRS as the reference standard, indicating that a Dixon-based technique could be useful for quantifying the hepatic fat fraction in the entire liver parenchyma.

To date, MRS has been used as the gold-standard noninvasive method for the detection and quantification of hepatic fat, with accuracy approaching or equaling that of liver biopsy^(26-29)^. However, MRS may not be available at all facilities and, more importantly, provides information only from a single voxel. This is a major limitation, given that hepatic fat distribution may be heterogeneous and sampling only a small part of liver can result in overestimation or underestimation of the hepatic fat content. In addition, if MRS is used in the follow-up patients under treatment, it may be difficult to replicate the sampling location exactly, which could limit the value of serial changes in the fat fraction. Dixon-based imaging may be reliably used to detect hepatic fat fraction in the entire liver parenchyma, as has been reported by other researchers, including Kühn et al.^(30)^, Idilman et al.^(31)^, Bhat et al.^(32)^, and Kang et al.^(33)^. In a meta-analysis, Qu et al.^(34)^ found that the MRI-PDFF has high diagnostic accuracy for the detection and quantification of hepatic fat when liver biopsy is used as the reference standard. We found multi-echo Dixon to have advantages over MRS, including the fact that it can evaluate the entire liver parenchyma and is relatively more widely available. Multi-echo Dixon has potential utility in the detection and quantification of hepatic fat in living liver transplant donors, rendering invasive technique such as liver biopsy unnecessary for hepatic fat quantification.

The unique aspect of our study was the evaluation of adults with no known liver disease. Most prior studies have evaluated patients with known or suspected liver disease, in whom biopsy could be performed. Recruiting participants with no known liver disease prevented us from performing liver biopsy on ethical grounds, limiting our focus to comparing the hepatic fat fractions derived by multi-echo Dixon and MRS.

The main limitation of the present study was the small sample size, which was mainly due to the relative short study period. In addition, because liver biopsy could not be performed, we had no gold standard for comparison. Furthermore, the possibility that some participants had viral liver disease or mild diabetes mellitus could not be completely ruled out.

## CONCLUSION

The diagnostic accuracy of multi-echo Dixon was found to be comparable to that of MRS, with a very strong positive correlation and good agreement between the hepatic fat fraction obtained by multi-echo Dixon (in an ROI and in the entire liver) and that obtained by MRS. This indicates that multi-echo Dixon could be used as an alternative to MRS when the latter is unavailable or when it is necessary to quantify fat in the entire liver parenchyma.
